# Phenolic Compounds and Flavonoids in Various Extracts of *Ferula tadshikorum* and *Ferula sumbul* Grown in vitro Conditions

**DOI:** 10.5812/ijpr-163731

**Published:** 2025-11-30

**Authors:** Dilafruz Jamalova Ne'matilla Qizi, Gulsauir Kurbaniyazova Tanirbergen Kizi, Imene Caliscan Tatar, Atoeva Rukhsora Odilovna, Nargiza Achilova Tuxtanazarovna, Azima Saitova Kaljanovna, Rabiga Esemuratova Xoshmuratovna, Boon Chin Tan, Azizbek Aliyor oʻgʻli Togʻayev, Ulugbek Kodirov Hamrokulovich, Ziyoviddin Yusupov Olimjon Ugli

**Affiliations:** 1 Institute of Botany of Academy of Sciences of the Republic of Uzbekistan, Tashkent, Uzbekistan; 2 Institute of Biological Sciences, Faculty of Science, Universiti Malaya, Kuala Lumpur, Malaysia; 3 Department of Biology, Faculty of Natural Sciences and Agrobiotechnology, Bukhara State University, Bukhara, Uzbekistan; 4 Jizzakx Politechnical Institute, Jizzakh, Uzbekistan; 5 Karakalpak State University Named After Berdakh, Nukus, Uzbekistan; 6 Centre for Research in Biotechnology for Agriculture (CEBAR), Universiti Malaya, Kuala Lumpur, Malaysia; 7 Termez University of Economics and Service, Termez, Uzbekistan

**Keywords:** Flavonoids, in vitro, p-Coumaric Acid, Kaempferol, Medicinal Plants

## Abstract

**Background:**

Flavonoids, which are bioactive compounds found in medicinal plant extracts, are recognized for their significant therapeutic properties. To efficiently isolate these compounds from fresh plant materials, it is essential to optimize extraction methods. The most widely used approach involves solvent extraction using solvents such as methanol, ethanol, acetone, and hexane. This study aimed to determine the optimal extraction methods for isolating phenolic compounds and flavonoids from callus and regenerating plants of two medicinal species of the genus *Ferula* cultivated in vitro with or without indole-3-butyric acid (IBA) and 6-benzylaminopurine (BAP). This study focuses on *Ferula tadshikorum* and *Ferula sumbul*, with extracts analyzed quantitatively using high performance liquid chromatography (HPLC). The results revealed the presence of flavonoids such as kaempferol, quercetin, p-coumaric acid, and cinnamic acid at varying concentrations across different solvents. Notably, extracts from *F. sumbul* plants grown with Murashige and Skoog (MS) medium containing IBA and BAP exhibited higher levels of p-coumaric acid (9.588 µg/mL) in methanol, and kaempferol (9.595 µg/mL) in hexane. For *F. tadshikorum*, ethanol extraction yielded a significant amount of p-coumaric acid (29.9 µg/mL). Quercetin and cinnamic acid were detected in minimal quantities in both extracts, whereas kaempferol was notably higher (2.61 µg/mL) than in the methanol extract (0.617 µg/mL).

**Objectives:**

The present study represents the first attempt to determine the phenolic compounds in regenerating plants obtained through in vitro methods.

**Methods:**

In vitro regenerated plants of *F. tadshikorum* and *F. sumbul*, cultured on MS basal medium supplemented with IBA (0.5 mg/mL) and BAP (0.5 mg/mL), were ground into a fine powder in liquid nitrogen and then dried in a drying oven at 50°C before chemical analyses. The bioactive compounds were extracted by soaking the powders in methanol, ethanol, and hexane overnight. The extracts were filtered and evaporated under a vacuum at 35 - 36°C. The resultant slurry was partitioned with equal volumes of ethyl acetate (EA) and water to remove excess polar compounds. The EA fraction was vacuum-dried, and the mass of the crude extract was recorded. The extract was subsequently dissolved in methanol, ethanol, and hexane to achieve a concentration of 1 mg/mL. Finally, the solution was filtered through a 0.45 µm PTFE filter (German Acrodisc 13 CR) before analysis by HPLC.

**Results:**

We then analyzed methanol, ethanol, and hexane extracts from in vitro *F. tadshikorum* and *F. sumbul* plants grown under various combinations of hormones and hormone-free conditions using HPLC. When comparing extracts prepared with 3 solvents from 2 different samples of the *F. sumbul*, plants obtained in a combination of IBA and BAP hormones showed a higher content of p-coumaric acid in methanol extraction (9.58 µg/mL) and a higher content of kaempferol in the hexane extraction (9.59 µg/mL). During the extraction of callus and plant regenerants from in vitro *F. tadshikorum* plants using methanol and ethanol, a higher content of p-coumaric acid (29.9 µg/mL) was observed in the ethanol extract than in methanol. Quercetin and cinnamic acid were detected in very low quantities in both the extracts. Notably, kaempferol content was higher (2.61 µg/mL) than in the methanol extract (0.617 µg/mL).

**Conclusions:**

This study focused on the declining wild medicinal herbs in natural populations, providing an opportunity for systematic in vitro propagation year-round. This approach aims to establish these herbs as alternative sources for extracting biologically active secondary metabolites such as flavonoids.

## 1. Background

*Ferula* L., a medicinal plant species, is rich in phenolic compounds, coumarins, terpenoids, flavonoids, and essential oils, all of which demonstrate notable biological activities (1-4). Among these species, *Ferula tadshikorum* Pimenov, native to Uzbekistan and Tajikistan, is particularly diverse in its medicinal properties. Its essential oils possess mild antioxidant and antimicrobial activities, similar to those found in other sulfur-containing *Ferula* oils (5). This plant is recognized for its expectorant and anticonvulsant effects, particularly under conditions such as exudative diathesis, pulmonary tuberculosis, otitis media, and lymphadenitis (6). *Ferula* species are traditionally used to treat vitiligo, tuberculosis, joint pain, parasitic infections, gastrointestinal inflammation, and as antidotes for toxic substances. In Central Asia, gum resin is used as an anthelmintic, insecticidal, and anticonvulsant (7), as well as for treating certain nervous diseases and viral infections (8) of the reproductive system. *Ferula sumbul* (Kaufm.) Hook. f., common in Central Asia (Uzbekistan, Tajikistan), remains relatively understudied in terms of flavonoid composition. Phytochemical screening of various root extracts has revealed the presence of triterpenoids, flavonoids, coumarins, phenols, alkaloids, proteins, and carbohydrates (9). Its roots are traditionally used to relieve anxiety, serve as a sedative for stress and neurosis, provide relief for hysteria and other nervous disorders, and act as mild gastrointestinal stimulants (9, 10). Additionally, they are employed in treating kidney and stomach diseases (8).

A group of polyphenolic biologically active compounds (BAC), including flavonoids, exhibits a diverse range of pharmacological actions. Flavonoids, such as flavonols, flavones, and anthocyanins, are secondary metabolites produced by plants in response to environmental stresses such as cold, drought, heat, salinization, ultraviolet radiation, and pathogenic microorganisms (11, 12). Recently, there has been an increase in the number of pharmacopoeial plants containing flavonoids and other polyphenolic compounds, highlighting the importance of phytochemical research and the search for new plant sources with significant pharmacological effects (13).

Quercetin and kaempferol are among the most prevalent flavonoids in plants and are known for their wide-ranging biological activities, including antioxidant, antitumor, anti-inflammatory, and anti-allergic effects (14). In cancer research, nanoquercetin has been shown to activate apoptosis in defective MCF-7 cells. Notably, quercetin can enhance the sensitivity of MCF-7 cells to the cytostatic anthracycline antibiotic doxorubicin, potentially overcoming drug resistance (15). Additionally, a treatment approach for gastric cancer has been developed using quercetin, which activates apoptosis in gastric adenocarcinoma cell lines (14). Kaempferol is a flavonoid produced in plants and is distinguished from quercetin by the absence of one hydroxyl group in its aryl structure (16). It exhibits anti-inflammatory effects through multifaceted actions on the mechanisms underlying the inflammatory processes. Kaempferol inhibits the synthesis of nitric oxide and the activity of hyaluronidase, collagenase, 15-lipoxygenase, and both cyclooxygenases (17).

Cinnamic acid and p-coumaric acid are precursors of pinocembrin and naringenin, respectively. Both flavonoids have recently garnered attention owing to their antimicrobial, antioxidant, antitumor, and anti-inflammatory properties (18). p-Coumaric acid and its derivatives exhibit a wide range of bioactive properties, including antioxidant, antimicrobial, anticancer, anti-arthritic, anti-inflammatory, gout prevention, anti-diabetic, anti-melanogenic, skin regeneration, gastroprotective, anti-ulcer, cardioprotective, hepatoprotective, renoprotective, bone formation, anti-angiogenic, and anti-platelet properties. Given its extensive bioactivity, p-coumaric acid has potential applications in edible food, pharmaceutical, and cosmetic products. However, further studies are required to evaluate the compatibility of these products. To the best of our knowledge, this is the first study to discuss the natural occurrence, extraction, natural derivatives, synthesis of various derivatives, and therapeutic applications of p-coumaric acid (19).

## 2. Objectives

The present study aimed to isolate and quantify biologically active substances from in vitro cultures of *F. tadshikorum* and *F. sumbul* by dissolving them in various solvents. Because these species primarily contain phenolic compounds, coumarins, flavonoids, and secondary metabolites related to terpenoids, the extraction methods focused on isolating substances from these groups. This study is the first to quantify phenolic compounds in in vitro-regenerated *Ferula* species.

## 3. Methods

### 3.1. Plantlet Cultivation

Plantlets of *F. tadshikorum* and *F. sumbul* were cultivated in vitro using Murashige and Skoog (MS) medium. The media were supplemented with 0.5 mg/mL indole-3-butyric acid (IBA), 0.5 mg/mL 6-benzylaminopurine (BAP), or maintained hormone-free. Cultures were incubated at 24 ± 2°C under a 16/8 h light/dark photoperiod with a light intensity of approximately 3000 lux. The plantlets were subcultured every 4 weeks to maintain vigor and growth.

### 3.2. Plant Materials

In vitro regenerated plants of *F. tadshikorum* and *F. sumbul*, cultured on MS basal medium supplemented with IBA (0.5 mg/mL) and BAP (0.5 mg/mL) (20), were ground into a fine powder in liquid nitrogen and then dried in a drying oven at 50°C before chemical analyses.

### 3.3. Extraction and Purification of Bioactive Compounds

For extraction, 500 mg of each plant sample was weighed and placed into flasks. An appropriate solvent was added (e.g., methanol), and the mixture was vortexed for 1 - 2 minutes. The samples were then sonicated for 5 minutes and stored overnight at 4°C. The next day, samples were centrifuged at 10,000 rpm for 10 minutes at room temperature. The supernatant was collected, and the residue was re-extracted by adding fresh solvent and repeating the vortexing, sonication, storage, and centrifugation steps. The combined extracts were evaporated under vacuum at 35 - 36°C until complete removal of solvent and water. Ethyl acetate (EA) and distilled water (1:1) were added to the residue and thoroughly mixed. The mixture was transferred to a separatory funnel and allowed to separate into two phases. The lower aqueous phase was discarded, and the upper organic phase containing flavonoids was collected. The organic phase was evaporated under vacuum again, and the residue was weighed. Finally, methanol was added in equal proportion to the residue mass, and the extract was stored refrigerated until further analysis. Finally, the solution was filtered through a 0.45 µm PTFE filter (German Acrodisc 13 CR) before analysis by high performance liquid chromatography (HPLC).

### 3.4. High Performance Liquid Chromatography Analysis

An injection volume of 20 µL was applied to each sample, and the eluent was monitored at 280 nm using an HPLC system (Shimadzu) equipped with a C18 column (Shim-pack C18, 5 µm, 4.6 mm × 250 mm) (Shimadzu, Japan). The system included a Shimadzu LC-20AT pump with a low-pressure gradient formation unit (LGPE), diode array detector (SPD-M20A), and Shimadzu Sil-20AHT autosampler operating at a flow rate of 1.5 mL/min. The solvent system consisted of (A) 0.5% (v/v) acetic acid in water, and (B) 0.5% (v/v) acetic acid in methanol. The elution gradient was as follows: 0 - 10% (B) (0 - 0.01 min); 20 - 60% (B) (0.01 - 2 min); 60 - 80% (2 - 15 min); 100% (B) (15 - 30 min); 100 - 10% (B) (30 - 35 min); and 10 - 0% (35 - 40 min). Each run lasted 40 min, and three runs were conducted for each sample. Only peak areas with a standard deviation of less than 5% were recorded. Quercetin (98 - 99% for HPLC, Sigma Aldrich, USA), kaempferol (98 - 99% for HPLC, Sigma Aldrich, USA), p-coumaric acid (0320-0595, Germany), and cinnamic acid (HWI Group, Germany) were used as standards (21).

### 3.5. Statistical Analysis

The experiments were conducted in triplicates and expressed as percentages. The data were analyzed statistically by analysis of variance (ANOVA) followed by Duncan’s multiple-range test at a significance level of P < 0.05.

## 4. Results and Discussion

Flavonoids are essential active compounds in medicinal plant extracts and offer significant health benefits (22, 23). Therefore, it is crucial to optimize the flavonoid extraction method to effectively isolate these compounds from fresh plant materials. The most commonly used method is extraction using solvents (methanol, ethanol, acetone, EA, chloroform, ether, and water) (24). Quercetin (hydrate) and kaempferol are soluble in organic solvents such as ethanol, dimethyl sulfoxide (DMSO), and dimethylformamide (DMF). Quercetin (hydrate) has a solubility of approximately 2 mg/mL in ethanol and 30 mg/mL in DMSO and DMF, respectively. In comparison, the solubility of kaempferol in these solvents is approximately 11 mg/mL in ethanol, 10 mg/mL in DMSO, and 3 mg/mL in DMF. As kaempferol is slightly soluble in water, it can also be extracted using a combination of organic solvents (mainly ethanol, methanol, and acetone). Similarly, cinnamic acid, a white crystalline compound, exhibits slight solubility in water but dissolves easily in various organic solvents such as benzene, diethyl ether, and acetone, while remaining insoluble in hexane (25). In this study, we focused on optimizing the extraction methods for quercetin, kaempferol, p-coumaric acid, and cinnamic acid. We then analyzed methanol, ethanol, and hexane extracts from in vitro *F. tadshikorum* and *F. sumbul* plants grown under different conditions, including IBA (0.5 mg/mL), BAP (0.5 mg/mL), and hormone-free MS medium (20) using HPLC (Table 1). 

**Table 1.** Retention Times and Concentrations of Compounds in *Ferula sumbul* Extracted Using Different Solvents ^a^

Peak	Standard	Formula	Methanol		Ethanol		Hexane	
			Rt (min)	Concentration (µg/mL)	Rt (min)	Concentration (µg/mL)	Rt (min)	Concentration (µg/mL)
**1**	Quercetin	C_15_H_10_O_7_	11.859	0.67 ± 0.02	11.846	0.526 ± 0.07	11.884	0.592 ± 0.05
**2**	Kaempferol	C_15_H_10_O_6_	36.453	0.465 ± 0.03	36.021	2.28 ± 0.47	35.588	9.52 ± 0.21
**3**	p-Coumaric acid	C_9_H_8_O_3_	9.435	9.58 ± 0.09	9.364	3.68 ± 0.2	9.432	0.65 ± 0.06
**4**	Cinnamic acid	C_9_H_8_O2	12.432	0.28 ± 0.02	12.315	0.13 ± 0.016	12.346	0.21 ± 0.017

Abbreviation: Rt, retention time.

^a^ Values are expressed as mean ± standard deviation.

**Figure 1.**
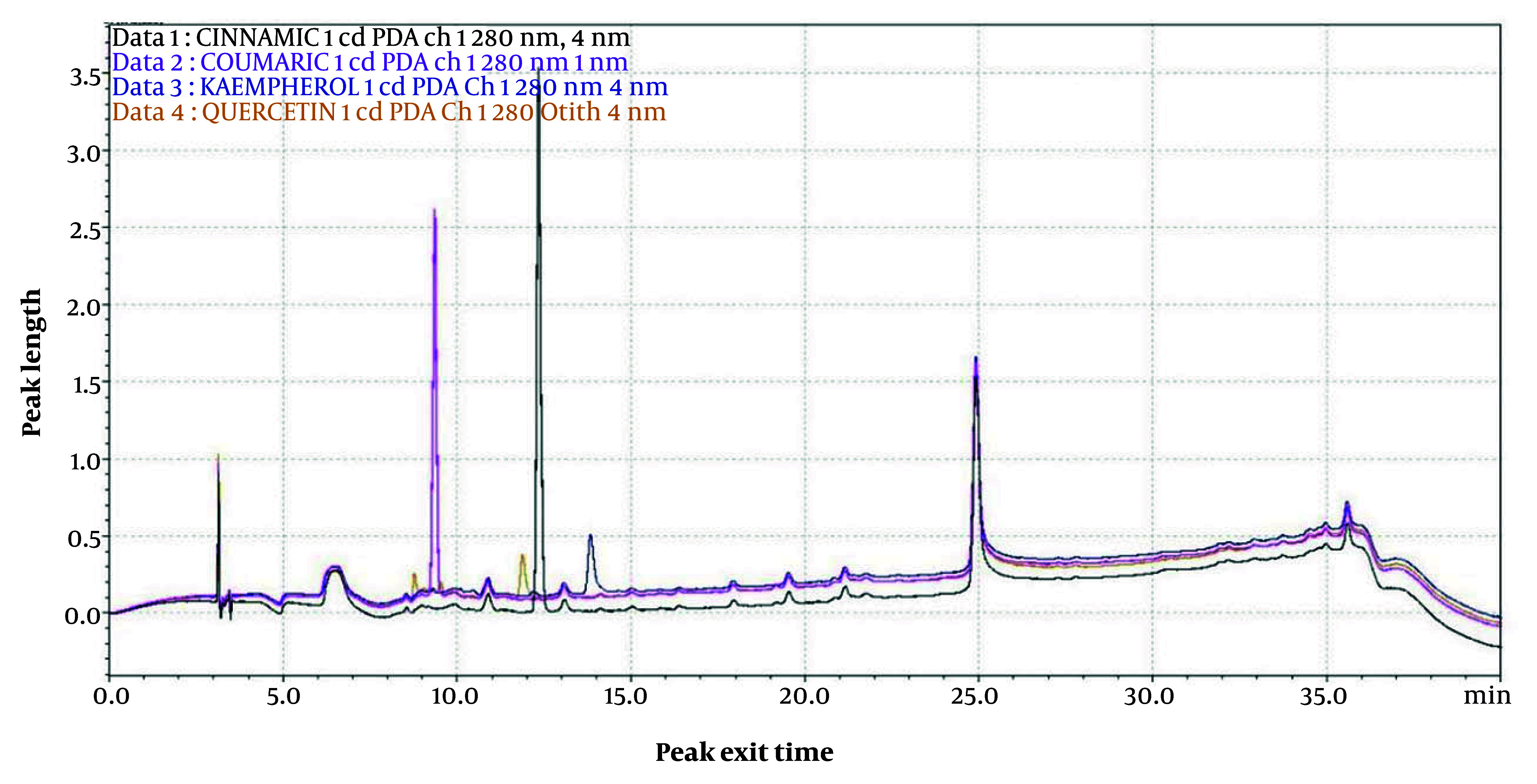
Chromatogram of standards (quercetin, kaempferol, p-coumaric acid and cinnamic acid)

We first determined the retention time (Rt) and peak profiles for four standards: Quercetin, kaempferol, p-coumaric acid, and cinnamic acid. The Rt values of quercetin, kaempferol at 36.453 min, p-coumaric acid at 9.435 min, and cinnamic acid were 11.859, 36.453, 9.435, and 12.432 min, respectively (Figure 1). 

**Figure 2.**
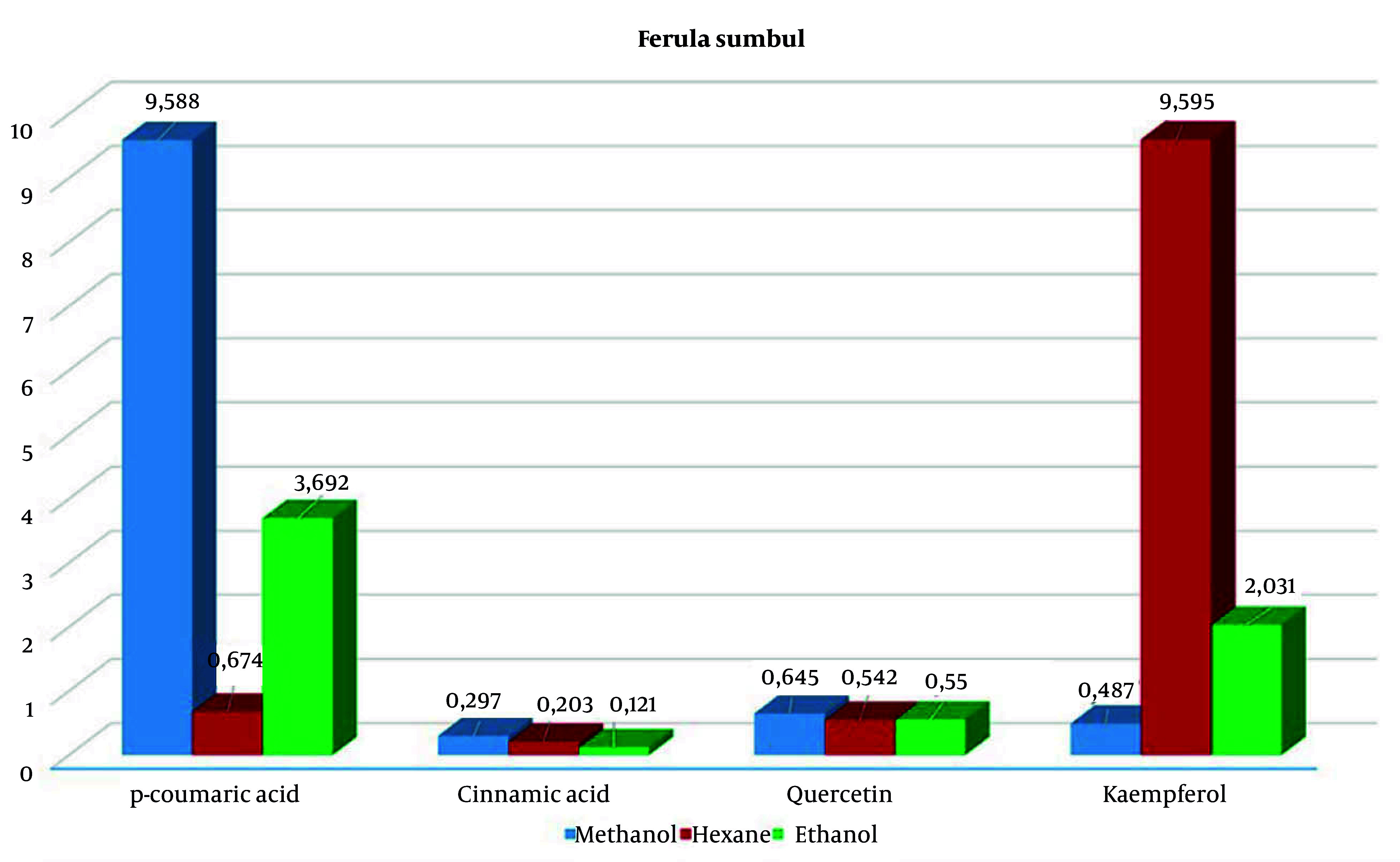
Statistical chart showing p-coumaric acid, cinnamic acid, quercetin and kaempferol concentrations in *Ferula sumbul* extracts.

When comparing extracts prepared with 3 solvents from 2 different samples of the *F. sumbul*, plants obtained in a combination of IBA and BAP hormones (26, 27) showed a higher content of p-coumaric acid in methanol extraction (9.58 µg/mL) and a higher content of kaempferol in the hexane extraction (9.59 µg/mL) (Figure 2). 

**Figure 3.**
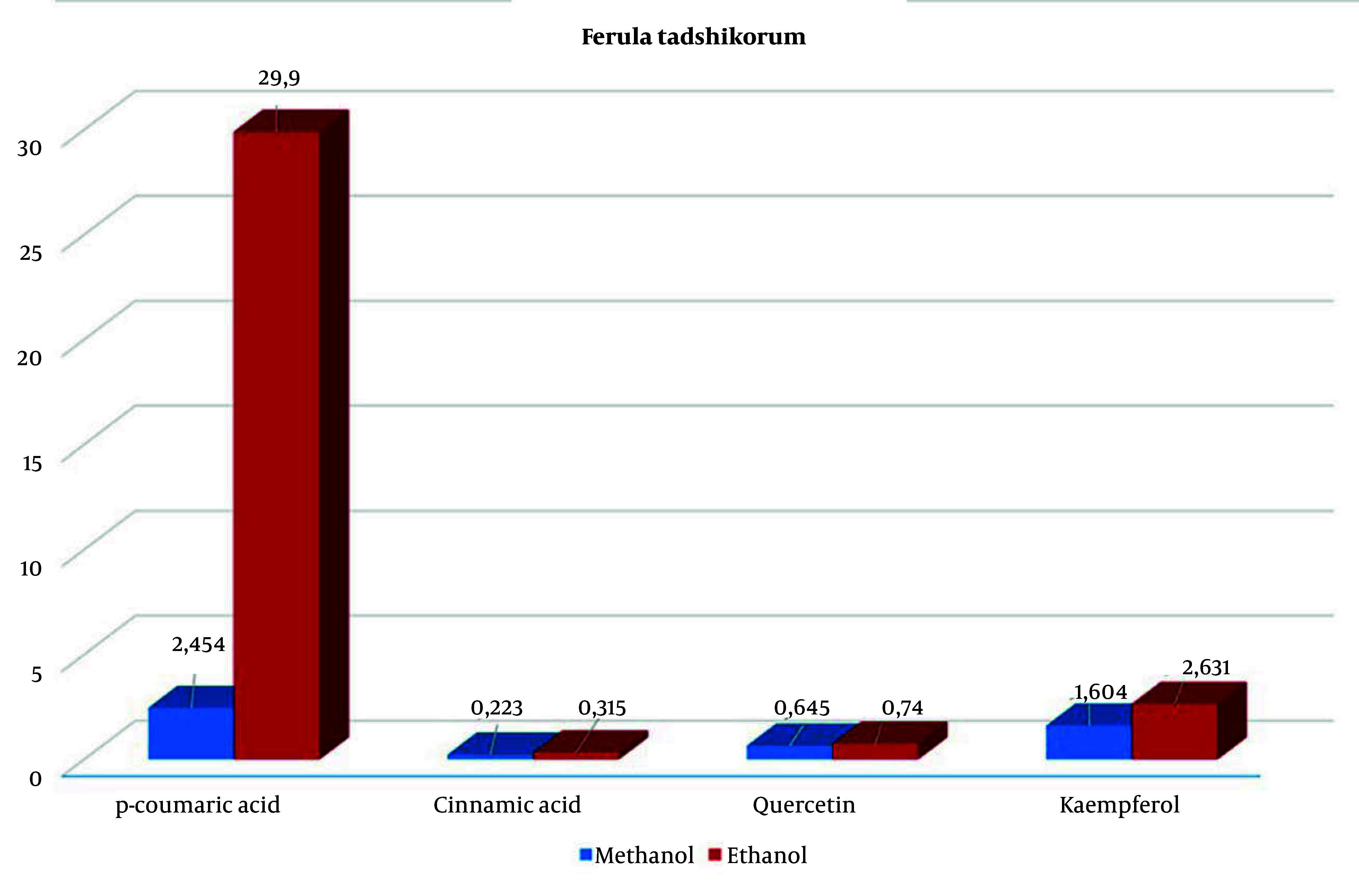
Statistical chart of compounds in *Ferula tadshikorum* regenerants extracted with methanol and ethanol

In contrast, plants grown in a hormone-free medium had lower levels of these compounds, with p-coumaric acid at 7.706 µg/mL and kaempferol at similar levels (Figure 2). However, the hexane extract contained significant amounts of cinnamic acid (4.09 µg/mL) and kaempferol (9.536 µg/mL). During the extraction of callus and plant regenerants from in vitro *F. tadshikorum* plants using methanol and ethanol, a higher content of p-coumaric acid (29.9 ± 0.64 µg/mL) was observed in the ethanol extract, compared to 2.44 ± 0.04 µg/mL in the methanol extract (Table 2). Quercetin (0.657 ± 0.03; 0.726 ± 0.07) and cinnamic acid (0.262 ± 0.04; 0.33 ± 0.003) were detected in very low quantities in both extracts. Notably, kaempferol content in the ethanol extract (2.63 µg/mL) was higher than in the methanol extract (1.604 µg/mL) (Figure 3). 

**Table 2.** Retention Times and Concentrations of p-Coumaric Acid, Cinnamic Acid, Quercetin, and Kaempferol in *Ferula tadshikorum* Extracted Using Different Solvents ^a^

Peak	Standard	Formula	Methanol		Ethanol	
			Rt (min)	Concentration (µg/mL)	Rt (min)	Concentration (µg/mL)
**1**	Quercetin	C_15_H_10_O_7_	11.859	0.657 ± 0.03	11.846	0.726 ± 0.07
**2**	Kaempferol	C_15_H_10_O_6_	36.453	1.604 ± 0.01	36.021	2.63 ± 0.32
**3**	p-Coumaric acid	C_9_H_8_O_3_	9.435	2.44 ± 0.04	9.364	29.9 ± 0.64
**4**	Cinnamic acid	C_9_H_8_O_2_	12.432	0.262 ± 0.04	12.315	0.33 ± 0.003

Abbreviation: Rt, retention time.

^a^ Values are expressed as mean ± standard deviation.

## 5. Conclusions

This study demonstrated that solvent type and in vitro culture conditions significantly influence the extraction efficiency of phenolic compounds and flavonoids from *F. tadshikorum* and *F. sumbul*. Among the tested solvents, ethanol yielded the highest concentration of p-coumaric acid in *F. tadshikorum* (29.9 ± 0.64 µg/mL), while hexane was the most effective for extracting kaempferol from *F. sumbul* (9.59 ± 0.21 µg/mL). Quercetin and cinnamic acid were detected in low concentrations across all samples. Plants cultured with hormonal supplementation (IBA and BAP) generally produced higher levels of bioactive compounds compared to hormone-free media. These findings suggest that optimizing solvent selection and hormonal conditions is crucial for maximizing the recovery of valuable phytochemicals from *Ferula* species. Furthermore, in vitro propagation of *Ferula* species offers a sustainable alternative to wild harvesting, helping to conserve natural populations that are increasingly threatened by overexploitation and habitat loss.

## Data Availability

All data is provided in the manuscript and in additional files. All data are available on request.
